# A Comprehensive Framework for Family-Centered Decision-Making in Perinatal Palliative Care: A Single Center’s Clinical Perspective

**DOI:** 10.3390/children13101379

**Published:** 2026-10-11

**Authors:** Licia Lugli, Marisa Pugliese, Elisabetta Garetti, Elisa Nieddu, Filomena Sileo, Camilla Migliozzi, Anna Ferrari, Francesca Culzoni, Natascia Bertoncelli, Alberto Berardi, Antonio La Marca, Emma Bertucci

**Affiliations:** 1 Neonatology and Neonatal Intensive Care Unit, Department of Medical and Surgical Sciences of Mothers, Children and Adults, University Hospital of Modena, 41100 Modena, Italy; alberto.berardi@unimore.it; 2 Psychology Unit, University Hospital of Modena, 41100 Modena, Italy; marisa.pugliese@unimore.it (M.P.); migliozzi.camilla@aou.mo.it (C.M.); ferrari.anna@aou.mo.it (A.F.); culzoni.francesca@aou.mo.it (F.C.); 3 Neonatology and Neonatal Intensive Care Unit, University Hospital of Modena, 41100 Modena, Italy; garetti.elisabetta@aou.mo.it (E.G.); nieddu.elisa@aou.mo.it (E.N.); bertoncelli.natascia@aou.mo.it (N.B.); 4 Prenatal Medicine Unit, Obstetrics and Gynecology Unit, University Hospital of Modena, 41100 Modena, Italy; sileo.filomena@aou.mo.it (F.S.); emma.bertucci@unimore.it (E.B.); 5 Obstetric and Gynecology Unit, Department of Medical and Surgical Sciences of Mothers, Children and Adults, Pediatric University Hospital of Modena, 41100 Modena, Italy; antonio.lamarca@unimore.it

**Keywords:** perinatal palliative care, life-limiting, life-threatening

## Abstract

**Highlights:**

**What are the main Findings?**

- We developed and implemented a structured perinatal palliative protocol through an internal multidisciplinary consensus process, in line with international perinatal palliative care guidelines. The protocol provides a comprehensive family-centered framework covering the entire care continuum—from antenatal diagnosis through bereavement.
- In a small pilot series, the protocol appeared feasible and adaptable to daily practice, although feasibility and adherence were not formally assessed. Observed challenges included prognostic uncertainty, inconsistent protocol application, and staff moral distress.

**What are the implications of the main finding?**

- The protocol offers a practical, reproducible model that other perinatal centers may adapt to help bridge the gap between guidelines and clinical practice.
- The structured framework is intended to support consistency, may help to reduce variability, and is designed to support shared decision-making that respects family values.

**Abstract:**

**Background:** Perinatal Palliative Care (PPC) is a specialized approach designed to support families facing a prenatal or postnatal diagnosis of a life-limiting or life-threatening condition. Despite the publication of authoritative international guidelines, a significant translational gap persists between aspirational recommendations and consistent, reproducible clinical practice. Structured and operational frameworks may contribute to PPC implementation in real-world clinical settings. This Perspective aims to provide a practical, operational instrument for PPC, derived from and aligned with international guidelines, that translates their principles into concrete clinical steps. **Methods:** We developed and implemented a structured PPC protocol at our tertiary academic center through a multidisciplinary review of existing PPC guidelines and an iterative internal institutional consensus process informed by clinical experience and feedback from parents and healthcare providers; this is a descriptive clinical Perspective rather than a formal research study. **Results:** The “Super Micky” PPC protocol provides a comprehensive family-centered framework incorporating: (1) clear eligibility criteria for PPC, derived from international guidelines and Italian recommendations; (2) multidisciplinary assessment integrating neonatology, obstetrics, psychology, nursing, and social work; (3) clear, empathetic, and continuous communication integrated with family values and priorities; (4) development of a personalized Palliative Birth Plan co-constructed with the family and reassessed after birth in the light of the infant’s clinical course; (5) ongoing emotional and psychological support; and (6) structured support for the clinical team through regular debriefings and ethical discussions. The protocol defines specific actionable steps across the entire care continuum, from antenatal diagnosis through labor, birth, neonatal hospitalization, and bereavement. Challenges observed in our clinical experience included prognostic uncertainty, variability in protocol application across clinical teams, and moral distress among staff. **Conclusions:** The “Super Micky” protocol is intended to translate the principles of family-centered care into a structured, practical instrument, derived from international guidelines, that other centers may adapt into routine perinatal practice; prospective evaluation of its feasibility, adherence, and family and staff outcomes is needed to confirm its impact. Its success depends on continuous team engagement, transparent communication with families, and robust support systems for healthcare professionals.

## 1. Introduction

Perinatal Palliative Care (PPC) is a specialized approach designed to support families facing a prenatal or perinatal diagnosis of a life-limiting or life-threatening condition. In this context, PPC emphasizes quality of life, symptom management, and holistic support, while remaining compatible with disease-directed or life-sustaining treatment when clinically appropriate. Grounded in a model that integrates maternal–fetal medicine, neonatology, psychology, nursing, social work, and spiritual care, PPC relies on multidisciplinary collaboration to guide families from the moment of diagnosis—delivering information with compassion, clarity, and honesty [1,2,3,4,5]. The need for appropriate care pathways is reinforced by a recent multicenter study [6], which reported a marked increase in continued pregnancies despite a prenatal diagnosis of complex and incurable fetal anomalies. Although palliative care in perinatal medicine has undergone considerable conceptual evolution in recent years, its integration into routine clinical practice remains variable across settings.

Despite the publication of authoritative international guidelines [7,8,9,10,11], on perinatal PPC from organizations such as the American Academy of Pediatrics (AAP) [7], the American College of Obstetricians and Gynecologists (ACOG) [8], the British Association of Perinatal Medicine (BAPM) [9], and the World Health Organization (WHO) [10], a significant translational gap persists. These guidelines articulate the principles of PPC—family-centered care, multidisciplinary collaboration, empathetic communication, and bereavement support. Several groups have begun translating these principles into dedicated care pathways, program-development guidance, and comprehensive care packages at the single-center or regional level [11,12,13]. However, few published models integrate the full continuum from prenatal counselling through bereavement into a single, step-by-step operational framework, and many centers still lack a structured tool for real-world implementation [7,8,9,10,11,12,14].

Consequently, translating broad recommendations into consistent and reproducible clinical workflows may remain challenging, particularly in complex clinical situations. This gap is particularly pronounced in the context of antenatally diagnosed life-limiting conditions, where the timeline is compressed, decisions are emotionally charged, and coordination between obstetric and neonatal teams is paramount. Furthermore, PPC may present a particular professional and ethical challenge for neonatologists, whose training and clinical practice are traditionally centered on life-sustaining treatment and the management of potentially reversible conditions.

PPC may therefore require clinicians to balance life-sustaining treatment with comfort, proportionality of interventions, and recognition of prognostic uncertainty. To address this implementation gap, we developed and implemented a structured PPC protocol at our tertiary academic center. This manuscript is presented as a clinical Perspective describing the development, structure, and implementation experience of this locally developed protocol. It is a descriptive account rather than a quality-improvement audit, formal guideline document, or research study with outcome data. This paper has two objectives: first, to describe our protocol—intended as a practical instrument for PPC derived from and consistent with international guidelines—and the operational steps we undertook; second, to share the challenges encountered in our clinical experience and lessons learned, providing a practical framework that other centers may adapt.

## 2. Developing the Perinatal Palliative Care Protocol

The development of the PPC protocol was guided by the aim of providing compassionate, coordinated, and continuous support to families facing a fetal or neonatal diagnosis associated with life-limiting or life-threatening conditions. Our PPC protocol was inspired by the first case managed through a dedicated comfort-care pathway at our center—an infant named Micky, born with a life-limiting condition. This experience highlighted to the clinical team the need for a structured and coordinated approach to supporting families facing similarly complex diagnoses. Therefore, this story contributed to the decision to formalize a protocol aimed at providing a consistent approach to coordinated, family-centered care. The protocol was developed through a multidisciplinary review of institutional experience, a review of feedback collected from parents and healthcare providers, and careful alignment with national and international PPC recommendations [7,8,9,10,11,12,14]. Throughout the manuscript, this feedback refers to a single, informal process: comments gathered from families and staff during the pilot phase and during team meetings, collated and discussed by the working group. It was not a structured survey, an audit, or a formal qualitative study.

The PPC protocol was formally established via an internal institutional consensus process that engaged the clinical stakeholders involved in perinatal care at our center. We adopted an iterative, Delphi-inspired approach—informed by, but not equivalent to, a formal Delphi study—in which successive rounds of discussion and anonymous feedback were used to build agreement on each protocol component. This iterative process supported progressive refinement and provided opportunities to discuss divergent viewpoints before institutional approval.

The process comprised the following sequential phases:

- Multidisciplinary working group: A core team was convened, comprising neonatologists, obstetricians, psychologists, midwives, and senior neonatal nurses. This group undertook a comprehensive review of the existing literature, international guidelines from the American Academy of Pediatrics (AAP), the American College of Obstetricians and Gynecologists (ACOG), the British Association of Perinatal Medicine (BAPM), the World Health Organization (WHO), the European Standards of Care for Newborn Health (EFCNI), and the Italian Recommendations for PPC, as well as published PPC models and institutional experiences [7,8,9,10,11,12,14].
- Iterative consensus-building through Delphi-like methodology: The working group held a series of structured meetings over several months. Between meetings, draft protocol components were circulated electronically, and anonymous feedback was collected from members. This iterative process—mirroring the core principles of the Delphi technique—allowed for the progressive refinement of each element, so that divergent viewpoints could be discussed within the group and the final content reflected the internal agreement reached by the multidisciplinary team.
- Pilot testing and refinement: The draft protocol was piloted on three clinical cases over a six-month period. Informal feedback (as defined above) was solicited from participating families and clinical staff, and this input was used to further refine the protocol’s structure, language, and documentation templates prior to full-scale implementation.
- Formal institutional approval: The finalised protocol underwent formal review by institutional leadership and received official approval, thereby enabling its integration into routine clinical practice. The protocol was formally named the “Super Micky” Protocol. This eponym serves not only as a lasting homage to Micky and his family but also as a constant reminder to our team of the core values that underpin the entire framework: compassion, dignity, family-centered care, and the unwavering commitment to honoring every infant’s life, however brief.

This process reflects an iterative institutional consensus-building method informed by clinical experience and elements of the Delphi technique, rather than a formal Delphi study with pre-specified participant numbers, rounds, or consensus thresholds; feedback gathered during the three pilot cases informed refinements to language, workflow, and documentation templates prior to full-scale implementation.

## 3. Structured Framework for Decision-Making in the “Super Micky” Protocol

PPC encompasses the continuum from antenatal diagnosis through labor, birth, neonatal hospitalization, and, when applicable, bereavement.

Unlike exclusively curative or life-prolonging care, PPC provides a spectrum of options ranging from comfort-focused care to concurrent palliative and disease-directed treatments [13,15,16,17,18,19,20,21]. Because perinatal life-limiting diagnoses involve high emotional and ethical complexity, structured guidance is essential. A structured family-centered framework is intended to support consistency and continuity in communication with families and to facilitate shared decision-making that respects parental values and priorities. Moreover, a structured framework may help reduce unwarranted variability in clinical practice and may provide a basis for addressing ethical challenges and emotional burden among healthcare professionals [22,23,24,25,26,27,28].

PPC plan should be documented clearly and reviewed regularly. In the “Super Micky Protocol”, we propose an effective PPC framework incorporating:

For clarity, this manuscript distinguishes three related but distinct concepts: (i) PPC consultation or integrated palliative support, which may be provided concurrently with disease-directed treatment; (ii) transition to predominantly comfort-focused care; and (iii) withholding or withdrawal of life-sustaining treatment, a separate decision made individually according to prognosis, the anticipated burden and benefit of treatment, the infant’s best interests, and family values. The conditions listed in Table 1 may reasonably prompt consideration of PPC but do not, by themselves, determine a decision to limit life-sustaining treatment; this distinction is maintained throughout Section 4, Section 5, Section 6 and Section 7.

- Clear eligibility criteria: PPC is indicated whenever a fetus or newborn is diagnosed with a condition that significantly limits life expectancy, threatens survival, or is expected to result in severe and irreversible morbidity (Table 1). Eligibility is determined by the overall prognosis, the anticipated burden of medical interventions, and the values of the family, rather than being tied to a single diagnosis. PPC may commence during pregnancy, immediately after birth, or later in the neonatal course as new clinical information emerges (Figure 1 and Figure 2). Because prognostic uncertainty is inherent to many of these conditions—both prenatally and, in some cases, even after birth—eligibility does not require certainty about outcome. Consistent with frameworks that explicitly recognize uncertainty in perinatal palliative care [9], a useful guiding question is whether the clinical team would be surprised if the infant were to die or to survive with severe morbidity; when the answer is no, initiating PPC consultation and parallel planning—preparing concurrently for divergent trajectories—is appropriate and does not preclude continued life-sustaining care where indicated.
- Multidisciplinary assessment: Neonatologists, obstetricians, nurses, psychologists, and other specialists collaborate to build a unified understanding of each case, ensuring that all medical, psychological, and social dimensions are addressed.
- Clear, empathetic, and continuous communication is integrated with family values and priorities: Information is delivered progressively, in accessible terms, and aligned with the family’s emotional readiness. At the same time, parental beliefs, hopes, and cultural or spiritual considerations are actively elicited and become essential elements of the care plan, ensuring that decisions reflect what matters most to the family.
- Development of a Palliative Birth Plan: A structured document is co-constructed with the family, outlining preferences regarding labor, neonatal comfort measures, early bonding, and end-of-life care. The birth plan is part of the clinical record and is reviewed regularly, with the understanding that it remains provisional and may be reassessed if changes in diagnosis or prognosis occur. Reassessment is undertaken systematically after birth and at defined points thereafter; care may be escalated as well as de-escalated as the clinical picture evolves, and the plan is never treated as a fixed determination of treatment limitation.
- Ongoing emotional and psychological support: Families receive continuous guidance before, during, and after birth, including dedicated grief and bereavement support, ensuring that their emotional needs are addressed throughout the entire care journey.
- Support for the clinical team: Regular debriefings and ethical discussions help to reduce fatigue, promote team well-being, and sustain the emotional resilience required to provide compassionate care in this challenging field [1,2,3,4,5].

## 4. Eligibility to the Super-Micky PPC Protocol

Eligibility for PPC is derived from international guidelines and Italian recommendations and is determined through a structured, multidisciplinary assessment [7,8,9,10,11,12,14]. PPC should be considered when a fetus or newborn is diagnosed with a condition associated with substantially limited life expectancy, significant risk to survival, or potentially severe and irreversible morbidity. Prenatally, eligibility is triggered when comprehensive diagnostic tools—including ultrasound, fetal MRI, and genetic testing—identify life-limiting conditions (e.g., anomalies incompatible with survival, severe structural defects with poor neurodevelopmental outlook, or uncertain but likely severe prognoses). Postnatally, PPC applies to confirmed life-limiting diagnoses, extreme prematurity at viability limits, severe complications, or chronic critical illness where treatment burden likely outweighs benefit (Table 1). Given diagnostic limitations, eligibility remains dynamic, requiring ongoing reevaluation as scientific evidence evolves. In this context, the terms extremely low birth weight (ELBW, birth weight <1000 g), extremely preterm (birth <28 weeks’ gestation), and periviable (approximately 22–25 weeks’ gestation, at the threshold of survival) describe overlapping but distinct populations and are not used interchangeably in this protocol; each requires individualized assessment. Across both the prenatal and postnatal periods, eligibility for PPC is grounded in the recognition that the burden of medical interventions may outweigh their potential benefits. Even when curative treatments are possible, PPC may be offered concurrently, ensuring that comfort, dignity, and family-centered support remain central. PPC is a new and evolving field in neonatology, and its principles may be applied to the care of ELBW infants. When palliative care management and family-centered care are utilized alongside standard medical management, they have the potential to significantly improve outcomes and the quality of life for both affected infants and their families. Extremely premature infants face multiple acute and chronic life-threatening conditions, and the treatments aimed at ameliorating or curing these conditions often entail significant pain and discomfort. By integrating palliative care from the very moment that extremely premature labor is diagnosed, families and clinicians receive crucial support through the process of defining goals of care and making difficult decisions about life support. For both the ELBW infant who dies soon after birth and the one who experiences multiple complications over weeks and months in the neonatal intensive care unit, palliative care can maintain a consistent focus on infant comfort and comprehensive family support, ensuring that dignity and compassionate communication remain at the forefront throughout the entire journey [29,30].

## 5. Family-Centered Decision-Making in PPC Super-Micky Protocol

At the Modena center, the clinical and relational approach is grounded in the principles of the Newborn Individualized Developmental Care and Assessment Program (NIDCAP) and the broader framework of Infant- and Family-Centered Developmental Care (IFCDC) [31,32,33,34,35,36]. The IFCDC model, originally designed to support neurodevelopmental outcomes in preterm and critically ill newborns, is extended to the PPC context, recognizing that every infant—regardless of prognosis—is a unique, sentient individual whose behavioral cues, physiological stability, and relational needs must be carefully observed, respected, and responded to throughout the entire care trajectory. Within this paradigm, decision-making is a dynamic, collaborative, and iterative process with families, while simultaneously protecting the newborn’s dignity, minimizing stress, and promoting gentle interaction.

From the moment of prenatal diagnosis, the care team initiates a “shared care planning” process, which represents the ethical and relational cornerstone of the entire palliative care journey. This is not a one-time discussion but an ongoing, evolving dialogue. Medical information is conveyed with clarity, honesty, and compassion, ensuring that parents fully understand their child’s condition, the range of possible outcomes, and the available options, without ever being overwhelmed by technical jargon or rushed into decisions. This shared journey naturally leads to the creation of a personalized birth plan—a document that translates the family’s values and preferences into concrete, actionable choices for the moment of birth. Crucially, the birth plan is not a rigid contract but a flexible guide, designed to adapt as circumstances evolve. As detailed in Section 3, the plan is reassessed systematically after birth, and care may be escalated as well as de-escalated as the clinical picture evolves. Every effort is made to balance what is clinically feasible with what the family hopes for, promoting early bonding and attachment through uninterrupted parental presence, gentle touch, and the creation of meaningful moments from the very first breath. Throughout this process, parents are offered quality time with their baby—unhurried, intimate moments that foster bonding and create lasting memories.

Postnatally, the IFCDC framework guides every aspect of care: through continuous, attentive observation of the newborn’s behavioral and physiological cues—including respiratory effort, neurological status, autonomic stability, motor tone, and pain responses—the multidisciplinary team adapts therapeutic interventions in real time to the infant’s changing needs, always prioritizing comfort, minimal handling, and protection from unnecessary stress. As outlined in Section 3, postnatal PPC may be provided alongside disease-directed and life-sustaining treatment. Where the goals of care have shifted mainly towards comfort-focused care, the primary aim is no longer to prolong life but to ensure comfort and quality of life while offering comprehensive support to the family; where disease-directed treatment continues, PPC is delivered concurrently with it. Symptom management includes prevention and treatment of pain, dyspnea, agitation, secretions, and feeding-related discomfort through individualized, proportionate pharmacological treatment titrated to adequate symptom relief while minimizing adverse effects, using non-invasive routes where possible, alongside essential non-pharmacological measures (skin-to-skin contact, gentle containment, swaddling, facilitated tucking, non-nutritive sucking, and a quiet, dimly lit, developmentally supportive environment). Respiratory support is guided by the current, regularly reassessed goals of care: within a comfort-focused approach, it is directed at the relief of distress, with oxygen therapy used when it alleviates symptoms, whereas invasive ventilation and intensive resuscitation are generally not undertaken unless they are consistent with the goals of care agreed with the family and reassessed at the time of the decision. Monitoring and diagnostic procedures are limited to those that directly contribute to comfort and decision-making, minimizing handling and disruptions to the infant’s rest-activity cycles.

Feeding decisions prioritize the newborn’s comfort and parents’ wishes, offering breastfeeding, expressed breast milk, or comfort feeding when feasible and well tolerated, while avoiding invasive nutritional support when it imposes a disproportionate burden; families are reassured that reduced intake at the end of life is a physiological process and not a source of suffering when appropriate comfort measures are in place. Concurrently, consistent with the IFCDC philosophy, parents are encouraged and supported in holding, touching, naming, and bathing, with unrestricted access whenever possible. Siblings and other family members may be involved according to parental preferences. This active involvement preserves the parental role and promotes attachment and bonding even within the limited time frame of palliative care [12,30,31,32,33,34]. Privacy and a calm, protected environment are ensured, particularly during end-of-life moments. Table 2 and Table 3 summarize the fundamental elements of the Super-Micky PPC protocol framework. Table 4 summarizes the main pharmacological agents recommended for pain control and sedation [37,38]. In the Appendix, we have reported the cases managed with the PPC protocol (Supplementary Table S1) [39], along with a narrative description of one of them (Supplementary Table S2); both are reported with the family’s written consent, as noted in the Informed Consent Statement. Supplementary Table S1 summarizes the 12 consecutive cases managed with the draft and final versions of the protocol, thereby describing the whole clinical experience accumulated at our center. It includes the three pilot cases—the first infants managed with the draft protocol during the six-month piloting phase, whose management was used to refine its wording, workflow, and documentation templates. Both the pilot cases and the remaining cases were reviewed retrospectively and descriptively, without predefined outcome measures, standardized data-collection forms, or assessment of protocol adherence.

## 6. Challenges of PPC Protocol Implementation

While the Modena protocol provides a structured framework for multidisciplinary collaboration, its completion has not been without challenges. In our clinical experience, we have observed several recurring difficulties that warrant transparent discussion. First, role clarity and coordination remain ongoing challenges. Despite clear definitions of team composition, the practical reality of busy clinical services often leads to overlapping responsibilities or, conversely, gaps in accountability. In our experience, the “continuity navigator” role—a senior nurse with dual training in neonatology and palliative care—has been essential for maintaining family-centered coordination, but this position requires dedicated protected time and specific training. Second, scheduling and attendance at multidisciplinary meetings pose logistical difficulties. Our protocol mandates periodic team meetings to review active PPC cases, but competing clinical demands frequently result in partial attendance. Third, consistency in protocol application may vary across clinical teams. While the referral triggers are predefined, some obstetric and neonatal colleagues remain hesitant to initiate palliative discussions early, particularly when prognostic uncertainty is high or when parents express strong preferences for life-sustaining interventions. This variability requires ongoing educational efforts and regular feedback on protocol adherence [1,2,3,4,5,6,7,8,9,10,11,12,14,29,30]. These observations derive from clinical experience, team debriefings, and informal feedback, and not from a formal evaluation of feasibility or adherence.

This experience has several limitations that should be considered when interpreting its applicability. First, it describes the protocol and implementation experience of a single tertiary academic center in Italy; direct generalizability to settings with different legal, financial, or organizational structures—including the availability of a dedicated continuity-navigator role—may be limited. Second, the protocol was piloted on a small number of clinical cases (three) prior to full implementation, which allowed initial refinement but does not provide a comprehensive picture of the diversity of situations that may arise with rarer conditions. Third, this manuscript is descriptive: it does not include a formal, structured evaluation of feasibility, protocol adherence, or family and staff outcomes following implementation; the cases summarized in the Supplementary Materials illustrate application of the protocol but do not constitute outcome data, and no control or comparison group was available. Prospective collection of such data—including family and staff satisfaction, adherence to protocol steps, and the diagnoses and number of families supported since approval—is planned and represents an important next step in evaluating and refining the Super Micky protocol. This prospective evaluation is intended to use predefined feasibility and adherence indicators, standardized data-collection forms, and validated family- and staff-reported outcome measures, in order to test whether the observations reported here are confirmed. Finally, legal, cultural, and healthcare-system factors shape PPC practice, and how Italian frameworks compare with those of other countries—for example regarding decision-making at the threshold of viability—merits dedicated discussion elsewhere [18].

Definitely, this single-center Perspective is descriptive: feasibility, protocol adherence, and family- and staff-reported outcomes were not formally assessed; no control or comparison group was available; and the sample was small. Accordingly, these findings should be interpreted as observations from clinical experience rather than results of a formal evaluation, and their generalizability to other settings remains uncertain.

## 7. Moral Distress in the Staff

The primary source of moral distress in PPC stems from the fundamental tension between the ingrained professional identity of the entire perinatal team—neonatologists, obstetricians, nurses, and midwives—all of whom are traditionally trained to sustain life—and the acceptance of death as a legitimate outcome. This conflict is acutely magnified when prenatal prognoses prove inaccurate. In cases where infants with severe life-limiting prenatal diagnoses exhibit unexpectedly favorable postnatal adaptation, staff and families may question whether the earlier focus on comfort care was premature or whether life-sustaining opportunities were inappropriately foreclosed, generating profound distress among team members who had participated in planning comfort-oriented care. Embracing prognostic uncertainty from the outset and openly discussing parallel plans for divergent outcomes with families and the team may reduce the likelihood that an initial comfort-focused plan is later perceived as premature (see Section 3). Conversely, when infants with less severe prenatal diagnoses experience sudden, unforeseen deterioration, staff grapple with the perception that families were inadequately prepared for the full range of possible outcomes, leading to feelings that expectations had not been met by the eventual course of illness. Beyond prognostic discrepancies, moral distress also arises when families request interventions that are unlikely to achieve their intended goal or decline recommended comfort measures, as well as when scientific evidence conflicts with local legal, religious, or cultural frameworks. To mitigate these challenges, our protocol incorporates structured debriefing sessions, ethical case discussions, transparent communication with families, and explicit acknowledgment of prognostic uncertainty from the outset. Regular case reviews allow us to adjust care plans dynamically when unexpected improvements occur, while revised communication strategies are intended to help families be better prepared for all possible trajectories [1,2,3,4,5,6,7,8,9,10,11,12,14,29,30,31,32,33,34].

## 8. Conclusions

The implementation of the “Super Micky” PPC protocol at our center represents a tangible response to the growing need for palliative care in the perinatal era. By translating the principles of family-centered care, multidisciplinary collaboration, and empathetic communication into a structured yet flexible framework, our experience suggests that PPC can be integrated into routine perinatal care, offering a practical instrument, derived from international guidelines, that other centers may adapt; prospective evaluation of feasibility, adherence, and family and staff outcomes is needed to confirm its impact. In our experience, the framework appeared feasible and adaptable, and it may help to support consistency and to reduce unwarranted variability in practice, although these effects were not formally measured. The challenges we encountered—ranging from prognostic uncertainty and variability in protocol application to the emotional toll on staff—have been instructive, prompting iterative refinements to our approach and reinforcing the need for ongoing education, debriefing, and ethical reflection.

## Figures and Tables

**Figure 1:**
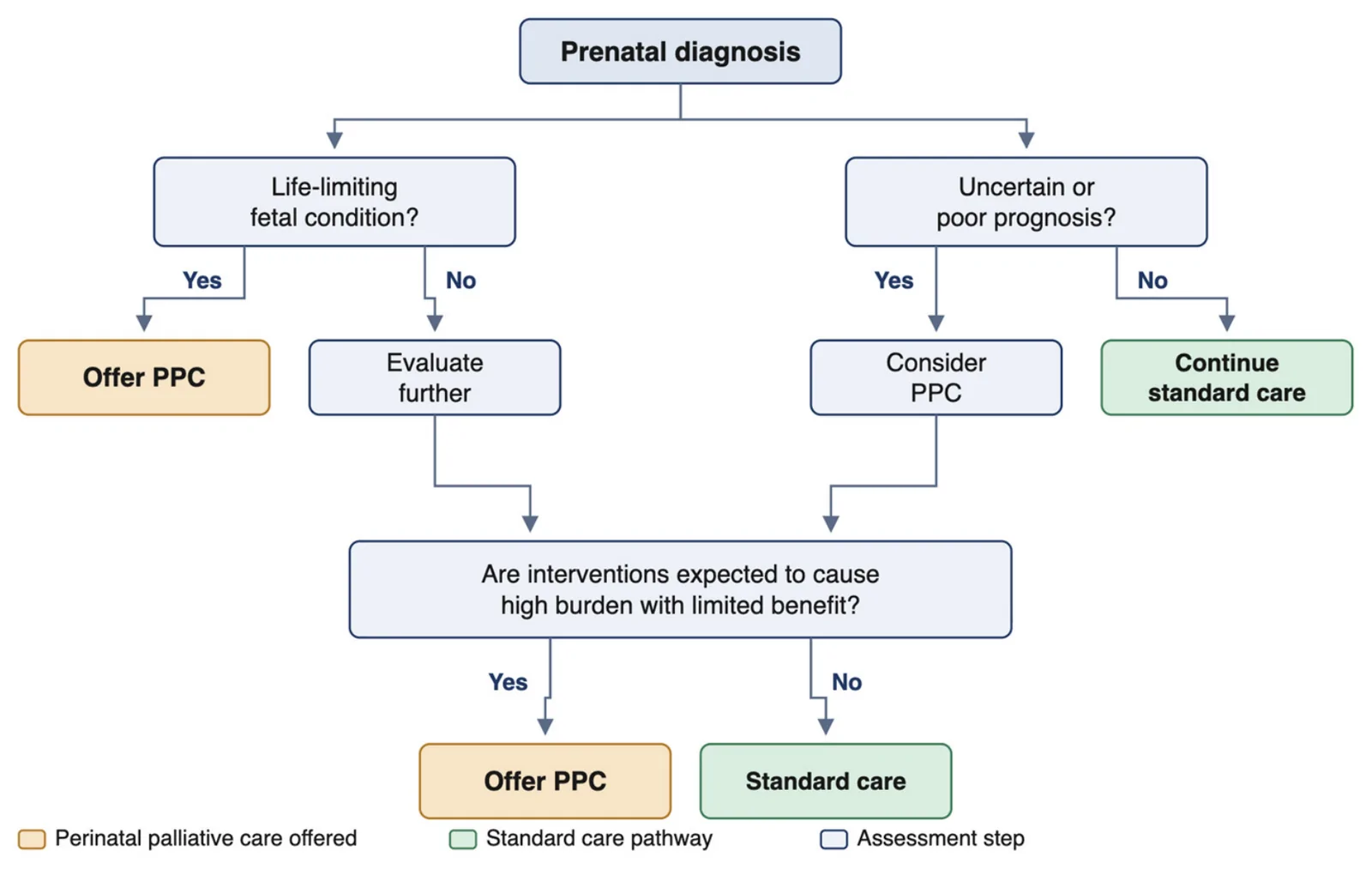
Prenatal Eligibility for Perinatal Palliative Care.

**Figure 2:**
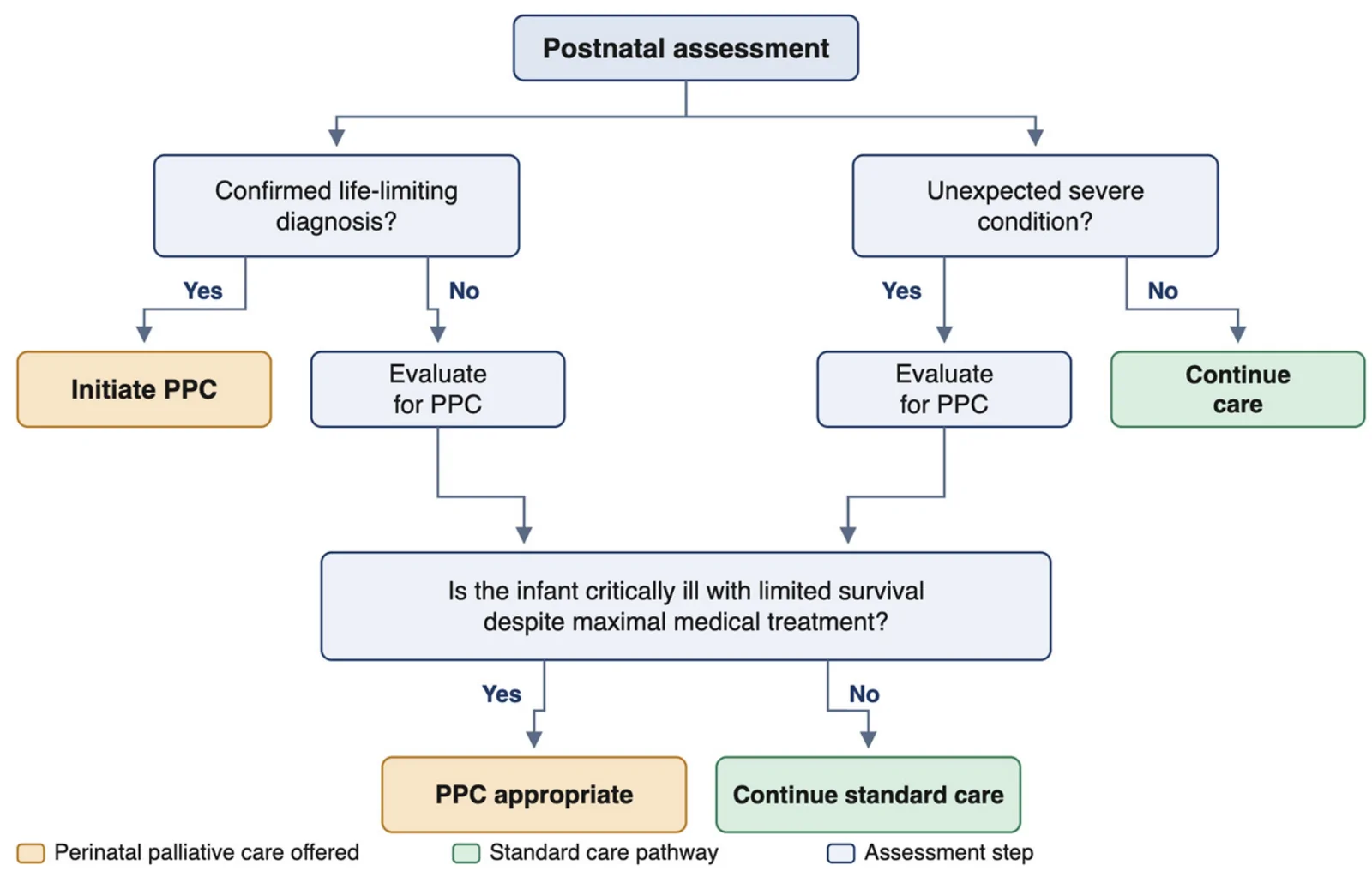
Postnatal Eligibility for Perinatal Palliative Care.

**Table 1:** Examples of Conditions Eligible for Perinatal Palliative Care (Prenatal and Postnatal) *.

Timing	Category	Subcategory	Examples of Eligible Conditions	Typical Prognosis/Rationale for PPC
Prenatal	Life-limiting congenital anomalies	Central nervous system	Anencephaly, alobar holoprosencephaly, severe lissencephaly	Incompatible with or severely limited survival; profound neurologic impairment
		Renal	Bilateral renal agenesis, severe renal dysplasia, multicystic kidneys with absent amniotic fluid	High mortality due to pulmonary hypoplasia
		Skeletal	Thanatophoric dysplasia, osteogenesis imperfecta type II	Chest hypoplasia → respiratory insufficiency
		Thoracic/Pulmonary	Severe congenital diaphragmatic hernia with liver herniation and low lung volume	Extremely poor postnatal survival
		Multi-system	Trisomy 13/18 with severe anomalies, body-stalk anomaly	Limited life expectancy; significant morbidity
	Severe conditions with poor or uncertain prognosis	Cardiovascular	Severe Hypoplastic Left Heart Syndrome variants not amenable to surgery, unbalanced AV canal, single ventricle with high risk	Multiple surgeries with high burden and uncertain outcome
		Neurologic	Severe ventriculomegaly, porencephaly, severe structural abnormalities	High risk of severe neurodevelopmental impairment
		Placental/fetal growth	Severe fetal growth restriction with absent/reversed end diastolic flow	High risk of stillbirth or neonatal demise
		Complex anomalies	Multiple congenital anomalies with unclear diagnosis	Unpredictable survival, high risk of major impairment
Postnatal	Life-limiting diagnoses confirmed after birth	Genetic/metabolic	Zellweger spectrum, mitochondrial disorders	Progressive, irreversible deterioration
		Cardiac	CHD not amenable to surgery, severe cardiomyopathy	Limited survival; high burden of intervention
	Severe neonatal complications	Hypoxic injury	Severe HIE with diffuse cerebral lesion	High mortality and profound neurologic impairment
		Prematurity	<22–23 weeks’ gestation	Survival extremely low; high morbidity
		Neurologic	Massive IVH with white matter injury	Severe disability expected
		Infectious	Fulminant sepsis with multi-organ failure	Poor survival despite maximal therapy
	Chronic critical conditions	Technology dependence	Long-term ventilation with no expected improvement	Very low likelihood of functional recovery
		Severe lung disease	End-stage bronchopulmonary dysplasia with cardiac/pulmonary failure	Progressive deterioration despite therapy

* Other conditions not reported in the table may be eligible for PPC. Conditions listed here may prompt consideration of PPC but do not, by themselves, determine a decision to limit life-sustaining treatment; eligibility and care decisions remain individualized and account for prognostic uncertainty (see Section 3).

**Table 2:** Perinatal palliative care protocol for neonates prenatally diagnosed with life-limiting conditions.

Phase	Recommended Actions
**During Pregnancy**	Provide prenatal counselling to communicate the diagnosis of a life-limiting or life-threatening condition eligible for perinatal palliative care (PPC). Engage a multidisciplinary team; conduct serial counselling sessions with the family and begin to define the individualized PPC plan. During counselling, explain comfort measures and reassure parents that optimal comfort for their baby can be achieved, regardless of the condition. Develop a birth plan collaboratively with the family, respecting their cultural, social, and religious preferences; provide a written copy to the family.
**Before Delivery**	Review and finalize the birth plan with the family. Prepare specialized dressings (e.g., for anencephaly, limb-body-wall complex) and appropriately sized clothing based on the baby’s condition and estimated size. If diagnostic confirmation requires postnatal blood testing, arrange for a cord blood sample at delivery.
**After Delivery**	Perform initial clinical assessment of the neonate. Apply a hat, place dressings as needed, and wrap the baby in a clean, warm blanket. Facilitate skin-to-skin contact and holding by the mother and/or father. Support bonding with siblings and extended family according to family wishes. Involve spiritual care services as per family preference. Capture photographs, videos, and memory-making items as desired by the family. Keep mother and baby together in the delivery room whenever possible or transfer to a private room. In selected cases where death is not imminent, consider transfer to the NICU.
**Care plan for the newborn and family**	Monitor vital signs (heart rate, respiratory rate, temperature) and pain scores (e.g., EDIN scale) as indicated by the baby’s clinical status. Avoid routine blood work or additional tests unless required for diagnostic confirmation. Encourage breastfeeding or gavage feeding as clinically appropriate; consider colostrum care for infants at end of life. Assess for signs of respiratory distress (e.g., air hunger, agitation, increased work of breathing, gasping). Implement non-pharmacological strategies (gentle upper airway suctioning, repositioning, non-nutritive sucking, sucrose) and/or pharmacological interventions (opioids or benzodiazepines) as needed to manage distress.

NICU: neonatal intensive care unit. EDIN scale: Échelle Douleur Inconfort Nouveau-Né [40].

**Table 3:** Palliative Care Protocol for Neonates with Postnatally Diagnosed Life-Limiting or Terminal Conditions in the NICU.

Domain/Phase	Recommended Actions
**Team Preparation & Shared Decision-Making**	Hold a multidisciplinary team discussion to review the baby’s clinical status and identify potential comfort-focused interventions. Communicate openly with parents to discuss the plan of care, shifting the goal toward quality of life and comfort. Collaboratively define a shared care program that aligns with the family’s values and preferences.
**Family-Centered Environment and Bonding**	Provide a private space within the NICU (single room, if available) to ensure confidentiality and emotional safety. Facilitate bonding through skin-to-skin contact, gentle holding, and gentle touch. Empower parents to participate in their baby’s care (e.g., colostrum administration, non-nutritive sucking, diaper changes, sponge baths, dressing). Include siblings and extended family in visits and bonding activities according to family wishes. Engage spiritual care services as requested by the family. Create memory-making opportunities (photographs, videos, hand/foot molds, keepsakes) as desired by the family.
**Clinical Management and Comfort Care**	Provide intermittent but readily available supervision by the medical and nursing team. When a transition toward comfort-focused care has been agreed upon, adjust or withdraw life-sustaining therapies according to the individualized goals-of-care plan; discontinue non-essential interventions when they no longer contribute to the infant’s comfort or agreed clinical goals. Silence monitor alarms and remove unnecessary devices to minimize environmental distress and promote a calm atmosphere. When an existing IV access is present, it may be maintained when clinically appropriate for symptom management and medication administration, while avoiding unnecessary invasive procedures. Continue to promote bonding and warmth through skin-to-skin care, holding, and colostrum administration. Regularly assess for signs of respiratory distress (e.g., air hunger, agitation, tachypnea, work of breathing, gasping). Use validated pain assessment tools (e.g., EDIN scale) to evaluate and monitor comfort levels. Implement non-pharmacological comfort strategies (positioning, gentle suctioning, soothing environment). Administer pharmacological agents as needed for pain and distress.
**Post-Mortem Family Support**	Following the baby’s death, offer parents the opportunity to bathe, dress, and spend time with their baby in a private, unhurried setting. Continue emotional and spiritual support for the family and provide information about bereavement resources and community support resources. Schedule a follow-up meeting with the family several weeks after the death (debriefing or restitution meeting) to review the care pathway together, share any pending diagnostic results, listen to the parents’ experiences, and offer further bereavement support.

NICU: neonatal intensive care unit. EDIN scale: Échelle Douleur Inconfort Nouveau-Né [40].

**Table 4:** Pharmacologic pain and sedation management.

Management	Drugs
Pain	Morphine Starting dose: 0.02–0.1 mg/kg IV or 0.2–0.4 mg/kg PO/SL q2–4h PRN Infusion: 0.02–0.1 mg/kg/h IV
	Fentanyl Starting dose: 1–3 mcg/kg IV/Intranasal q1–2h PRN Infusion: 1–3 mcg/kg/h IV
	Acetaminophen 10–15 mg/kg PO/IV or 20 mg/kg PR q6h
Pain and Sedation	Dexmedetomidine Starting dose: 0.5–1 mcg/kg IV or 2–4 mcg/kg PO/SL/buccal/intranasal q1–4h PRN Infusion: 0.2–1 mcg/kg/h IV
	Gabapentin 5–10 mg/kg PO q8–12h
Sedation	Midazolam Starting dose: 0.05–0.1 mg/kg IV or 0.2 mg/kg PO/SL/Intranasal q1h PRN Infusion: 1–2 mcg/kg/min IV
	Diazepam Starting dose: 0.05–0.25 mg/kg PO/PR q4–12h

PO: orally. IV: intravenous. PR: rectally. SL: sublingual. PRN = pro re nata. Doses are adapted from published neonatal end-of-life symptom-management guidance [37,38]. The intranasal routes of fentanyl and dexmedetomidine and the buccal/intranasal routes of midazolam are reported in the neonatal palliative care literature [37,38] but rest on limited neonatal and paediatric experience rather than licensed neonatal indications; they are therefore listed as off-label options, to be used with close monitoring and titration to effect. Routes not directly supported by the cited sources (oral/sublingual dexmedetomidine and oral/sublingual midazolam) have been removed from this table. Clinicians should consult institutional formularies and current pediatric palliative care references before prescribing, particularly for non-intravenous routes of fentanyl, midazolam, and dexmedetomidine, which warrant scrutiny in this population.

## Data Availability

No new data were created or analyzed in this study. Data sharing is not applicable to this article.

## Supplementary Materials

The following supporting information can be downloaded at: https://www.mdpi.com/article/10.3390/children13101379/s1, Table S1: Summary of Cases Managed Under the Super-Micky PPC Protocol; Table S2: Narrative report of PPC in neonate with Meckel-Gruber Syndrome prenatal diagnosis.
