# Supplementary material for: A Comprehensive Framework for Family-Centered Decision-Making in Perinatal Palliative Care: A Single Center’s Clinical Perspective
Source: Children (Basel). 2026 Oct 11;13(10):1379. doi: 10.3390/children13101379 (PMC13702993; doi:10.3390/children13101379)
Supplement: Supplementary file 1 [file children-13-01379-s001.zip › children-4541162-supplementary.pdf]

Supplementary Table S1. Summary of Cases Managed Under the Super-Micky PPC Protocol

Case Category	Diagnosis (n)	Key Interventions & Focus	Palliative Protocol
Prenatal Lethal (n=6)	- Anencephaly (2)<br>- Trisomy 18 (2)<br>-Meckel-Gruber syndrome (1)<br>- Apert syndrome + severe diaphragmatic hernia (1)	- Vaginal delivery at term<br>- Immediate skin-to-skin contact.<br>- Pharmacological comfort (oral sucrose, morphine, midazolam).<br>- Systematic memory-making (photos, footprints, hair locks).	"Comfort-First" Birth Plan
Postnatal Acute (n=4)	- HIE (asphyxiated term) (2)<br>- Asphyxiated preterm + Grade IV IVH (1)<br>- Preterm + severe PPHN (1)	-HIE: Redirected care after confirmation of severe brain injury (post-hypothermia).<br>-IVH: Comfort care after failed intensive care; continuous opioid infusion; minimal handling.<br>-PPHN: Gradual weaning from pressors, sedatives, and iNO; transition to skin-to-skin.	"Transitional Palliative Care"<br>(Withdrawal from active intensive therapies)
Chronic Supportive (n=2)	- Preterm infant with severe BPD, blindness, and cerebral lesions<br>- COG6 gene homozygous mutation (progressive neurological decline)	-Ongoing symptom management.<br>-Comfort measures and family accompaniment throughout the decline.	Quality-of-Life Focused Care

HIE: Hypoxic-Ischemic Encephalopathy. IVH: Intraventricular Hemorrhage. PPHN: Persistent Pulmonary Hypertension of the Newborn. iNO: inhaled nitric oxide. BPD: bronchopulmonary dysplasia. *COG6: Conserved Oligomeric Golgi Complex Subunit 6.*

Supplementary Table S2. Narrative report of PPC in neonate with Meckel-Gruber Syndrome prenatal diagnosis

**Prenatal diagnosis**
Meckel-Gruber Syndrome (MGS) was prenatally diagnosed. MGS is a severe, lethal, genetic disorder (autosomal recessive), characterized by a classic triad: occipital encephalocele, large cystic kidneys, and postaxial polydactyly
**Prenatal multidisciplinary counselling**
During the first trimester of pregnancy, prenatal imaging revealed multiple severe congenital anomalies consistent with MGS, including a large occipital encephalocele, postaxial polydactyly, and bilateral cystic kidneys. Prenatal genetic testing confirmed the diagnosis, identifying a pathogenic variant in the *TMEM231* gene, causative of MGS. Multiple counseling sessions were conducted. The diagnosis was discussed in detail with the parents by a multidisciplinary team—including obstetricians, neonatologists, and geneticists—during several dedicated meetings. The parents were informed that MGS is a rare, autosomal recessive ciliopathy characterized by lethal malformations. It was explained that the combination of encephalocele and cystic renal dysplasia is incompatible with long-term survival and is associated with severe neurological impairment, pulmonary hypoplasia, and renal failure. Survival beyond the neonatal period was described as extremely unlikely. Available options were discussed in a non-directive manner, including continuation of the pregnancy with perinatal palliative care planning and the option of pregnancy termination. The parents were informed that no curative or life-prolonging postnatal treatments would be available, and that neonatal care would focus on comfort, relief of distress, and family bonding.<br>The possibility of a comfort-focused birth plan was introduced, including skin-to-skin contact at birth, avoidance of invasive resuscitation, symptom control, memory-making activities, and support for cultural and religious preferences. The parents were reassured that the healthcare team would remain present throughout labor, birth, and the postnatal period. Throughout counseling, adequate time was given for questions and emotional support. The parents demonstrated understanding of the prognosis and expressed their values and wishes, which were respected and documented.
**Postnatal care**
S., a male infant, was born by spontaneous vaginal delivery. During labor, the mother was accompanied by her aunt and cousin; her husband, who was present in the hospital throughout, entered the delivery room periodically. At birth, the newborn presented cyanosis and bradypnea, with grunting and occasional gasping. Immediately after delivery, a cap was placed to cover his head and exposed brain, and—while still attached to the umbilical cord—he was placed in his mother's arms to ensure skin-to-skin contact. S. appeared calm, with no signs of pain or distress, although cyanosis, bradypnea, intermittent gasping, and grunting persisted. At the mother's request, the grandmother, assisted by the neonatal nurse, washed and dressed S., after which he was returned to his mother's arms. Approximately one hour after birth, S. began to show signs of discomfort, with increased grunting resembling crying. In agreement with the mother and in accordance with the birth plan, the neonatologist and nurse placed a nasogastric tube and administered morphine, resulting in relief of discomfort. A second bolus was given about 90 minutes later for recurrence of similar symptoms. During the first hour of life, the mother and relatives made video calls to family and friends and took photographs with S. Over time, S. became progressively paler and increasingly difficult to keep warm. He remained in his mother's arms and was covered with sheets and a wool blanket prepared by the parents. After the birth, the father entered the delivery room only once and stayed briefly; the grandmother remained continuously present. The mother held S. throughout, caressing his face, holding his hands to keep him warm, and speaking to him alternately in Italian and Arabic. Three hours after birth, the neonatologist auscultated S. and noted a heart rate below 60 bpm, worsening bradypnea, absence of grunting, rare gasping, and no spontaneous movements, with only occasional responses to stimuli such as the mother's voice and touch. Approximately ten minutes later, cardiac activity ceased. Post-mortem care was provided by the midwives, who—according to the mother's wishes—took additional photographs and obtained handprints and footprints to complete the memory box. Following S.'s death, the father returned to the delivery room. Throughout the time in the delivery room, the midwife and nurse were continuously present, while the on-call neonatologist alternated between the delivery room and the neonatal ward. The following day, the nurse who had attended the birth and two neonatologists who had known the mother during pregnancy visited her in her hospital room. The mother warmly welcomed them and expressed gratitude for the care provided.

TMEM231:Transmembrane Protein 231
